# Violating the salary cap: exploring performance gains in the National Basketball Association

**DOI:** 10.3389/fspor.2025.1625458

**Published:** 2025-07-28

**Authors:** Sean Pradhan, Dima Leshchinskii

**Affiliations:** School of Business, Menlo College, Atherton, CA, United States

**Keywords:** salary cap, luxury tax, professional basketball, team performance, roster construction

## Abstract

Salary caps, which act as price ceilings for teams on the cost of players, are commonplace in various North American professional sports leagues. Although some leagues have “hard” caps that teams cannot surpass (e.g., the National Football League), the National Basketball Association (NBA) utilizes a more flexible framework known as a “soft” cap, where the cap can be exceeded by paying a luxury tax, or penalty fee. Thus, teams can choose to optimize player salaries within the cap or strategically exceed it, if the marginal benefits outweigh the cost of the luxury tax. The purpose of the current study is to determine if violating the salary cap in the NBA warrants the financial burden associated with the luxury tax. Salary cap data spanning the 2011–2012 to 2023–2024 NBA seasons were collected from Spotrac, while team performance data were obtained from Basketball-Reference. Using each team's estimated luxury tax bill, we categorized teams into either those that violate the cap (violators) or those that do not (non-violators) based on each season. On-court performance and playoff status (playoff vs. non-playoff team) are compared using a series of mixed-effects models with random effects for team and season controlling for market size, operationalized using the population of the Census-defined metropolitan area, and the roster's average age. We test the hypothesis that paying for more expensive rosters justifies financial fines for violating the salary cap due to the ensuing improvement in team performance.

## 1 Introduction

In order to promote competition among member teams, a variety of professional sports leagues in North America use a salary cap system. The league sets an amount that each team can spend on its players' salaries, and teams cannot exceed this amount. However, the cap can be “hard”, or it can be “soft,” as in the National Basketball Association (NBA). NBA teams are able to violate the salary cap, but they must pay penalty fees in the form of a luxury tax for doing so. If the benefits of violating the salary cap—improved team performance, increased revenues, etc.—exceed the penalty paid, then a team might be interested in making such a violation. However, there are some exceptions to this system like the Qualifying Veteran Free Agent Exception or “Larry Bird” Exception, which allows teams to surpass the salary cap when re-signing players up to a specified maximum amount without incurring financial penalties, further increasing the complexity of the salary allocation process. During this study, we advance and test the hypothesis that paying for more expensive rosters justifies financial fines for violating the salary cap due to the resulting gains in team performance.

Our research follows the strands of literature that study the relationships between salary and team performance in North American professional sports and what effect salary concentration has on team performance. We selected the NBA for our study because earlier research provides some evidence that increases in salary concentration are associated with greater win percentages in the NBA compared to other professional sports leagues (1). Our work extends a prior study on salary cap violations in Australia (2) to the context of salary cap violations in the NBA.

## 2 Literature review

The link between salary and team performance in North American professional sports, as well as the question of optimal salary structure in terms of its concentration among top players has long been a subject of academic studies. With respect to the latter, Lazear and Rosen present a model in which heterogeneous and observable players' quality leads to a variation in salaries as an incentive to optimal allocation of resources (3). Similarly, Rosen, in his study of the “superstar” effect, shows that “[i]n certain kinds of economic activity there is concentration of output among a few individuals, marked skewness in the associated distributions of income and very large rewards at the top” (4). Simmons and Berri also conclude that increased pay inequality enhances player performance in the NBA and increases the probability of team success (5).

The effect of the salary cap system in North American professional sports on teams’ performance and the popularity of respective sports leagues have been a topic of discussion since the introduction of the salary cap within the NBA in 1983. In their book, “Pay Dirt: The Business of Professional Team Sports”, Quirck and Fort mention that although NBA team owners argued that “a salary cap was essential if ‘competitive balance’ was to be preserved in a league [NBA]… [t]he evidence suggests that the success of the NBA was due to the general increase in interest in sports during the 1980s” (6). Nevertheless, several studies provide support to the claim that salary cap implementation increases social welfare by narrowing the gap between NBA teams (7–9).

On the other hand, Katayama and Nuch examined game-level NBA data and find no link between team performance and salary distribution (10). It is possible the optimal salary distribution is sport dependent. Frick et al. assess the effects of wage distribution in the National Football League (NFL), NBA, Major League Baseball (MLB), and National Hockey League (NHL) (1). They conclude that increases in concentration are associated with higher win percentages in the NBA than in other leagues. For example, in the case of MLB, Tao et al. use data from 1985 to 2013 and find that greater wage disparity is negatively related to team performance (11). In their study of the NFL, Quinn et al. find that teams in the NFL have a “superstar” salary structure, with some players earning far higher salaries than others (12). These findings are supported by other studies. Using data from the 2000 to 2009 NFL seasons, Zimmer focuses on the assessment of how payroll distribution affects team performance, determining that salary concentration has a non-linear influence. That is, acquiring elite talent is likely the best alternative to achieve high levels of team performance (13). In their study of the NFL, Mullholland and Jensen identify which positions are worthy of greater investment. Using a combination of univariate regression models, they conclude that “it is worth investing in elite players at the quarterback, guard, defensive line, and linebacker positions” (14).

Other studies have looked at the possible connection between the impact of salary on overall team performance. Longden studied the effect of salary cap violations in the Australian National Rugby League between 2001 and 2010, and found that periods of cap violations by three rugby teams coincided with significant improvement of their performance, although this could be attributed to other factors (2). In a study of NHL teams, Glasnapp uses the Granger causality test on NHL payroll and performance data from the 1998–1999 to 2003–2004 seasons. However, this study did not find conclusive evidence that “supports the common belief that payroll can be used to predict a team's success” (15). In a similar vein, Lyons et al. attempt to identify an NBA player's performance variables that significantly contribute to determine that player's salary. They utilize multiple regression to analyze the 2013–2014 salaries of 243 NBA players and their career performance variables. Results indicated that points per game, rebounds, and personal fouls contributed significantly to a player's salary. Although the paper focuses on the salary distribution, it does not address the salary cap along with the luxury tax (16). Our study intends to address these gaps in the literature using data from the NBA.

## 3 Methodology

### 3.1 Data collection and variables

Team spending data were obtained from Spotrac (17), an online sports database containing information on team payrolls and player contract information from 2011 to the present. Data from the 2011–2012 to 2023–2024 NBA seasons were gathered for analysis. Using each team's estimated luxury tax bill, we categorized teams into either those that violated the salary cap (*violators*) or those that do not (*non-violators*) for each season. Team performance data for each season were gathered from Basketball-Reference (18), a publicly available sports database. Specifically, we examined season win percentage, whether the team made the playoffs (1 = playoffs, 0 = missed playoffs), as well as offensive rating and defensive rating. Offensive rating refers to the number of points a team produces for every 100 possessions (i.e., higher is better), while defensive rating involves the points a team allows per 100 opponent possessions (i.e., lower is better) (18).

### 3.2 Statistical analysis

All statistical analyses were performed in RStudio (19). First, we performed both standard and repeated-measures point-biserial correlations to examine the relationships between salary cap violation, main outcomes, and covariates. The repeated-measures approach was used to tease out variability within individual teams. Subsequently, we utilized mixed-effects models, which serve as an extension of linear regression to include both fixed and random effects. Fixed effects are analogous to the linear predictors from a standard linear regression, while the random effects are not directly estimated but are summarized according to their estimated variances and covariances. This structure offers additional flexibility to the statistical model, making it possible to model the random intercept and/or random slope as independent, correlated, or independent with equal variances (20). Many performance studies have used mixed-effect models to account for variability within and between groups (20, 21). In fact, McElreath contends that “that mixed models deserve to be the default form of regression” because they account for repeated sampling and sampling imbalance, model variation among individuals or groups, and avoid averaging (22).

### 3.3 Model specification

Differences among salary cap violators and non-violators for season win percentages, playoff status (playoff vs. non-playoff team), and the abovementioned on-court performance metrics were examined using a series of mixed-effects models with random effects for team and season. Linear models were specified for win percentage, offensive rating, and defensive rating. A binomial logistic model was utilized for advancement to playoffs due to the binary nature of variable. Across all models, we controlled for market size and the roster's average age. Market size was operationalized using the population of the Census-defined metropolitan statistical area (MSA) in Canada (23) and the United States (24). Previous research on men's and women's professional basketball has shown a significant linear and, in some instances, quadratic impact of player age on various performance outcomes (25–27). Therefore, we entered roster average age as both a linear and quadratic term in our models. Covariates were mean-centered for the analyses. For each model, we computed marginal and conditional *R*^2^ values. Marginal *R*^2^ refers to the proportion of variance explained by the fixed effects, while conditional *R*^2^ describes variance explained by the entire model [i.e., fixed and random effects; (28)]. To tease out the potential impact of extenuating circumstances, separate models were constructed that included all sampled seasons (i.e., 2011–2012 to 2023–2024) and those that excluded shortened seasons (i.e., the 2011–2012 season due to the NBA lockout and 2019–2020 season due to the COVID-19 pandemic). As a sensitivity analysis, we also evaluated identical models that excluded outliers in win percentage. Thus, teams with win percentages below the 5th percentile or above the 95th percentile were excluded from these analyses. Pirateplots were used to present results (29). This type of plot offers a visualization of the raw data points, central tendency (median), first and third quartile, and density.

## 4 Results

### 4.1 Summary statistics

**Figure 1:**
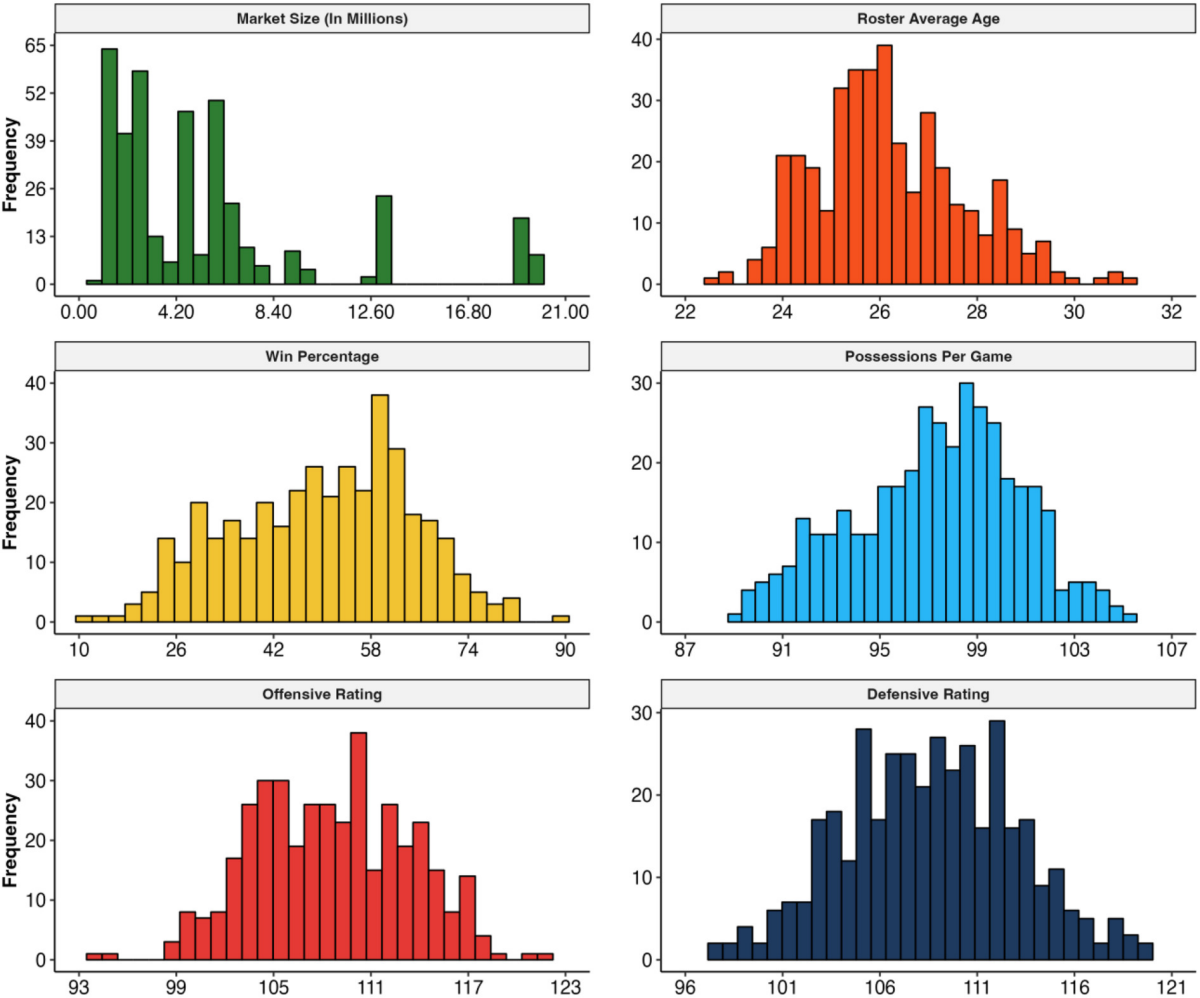
Histograms of sample statistics.

Data from 390 NBA team-seasons were featured in our sample (30 unique teams across 13 seasons), with 78 teams categorized as salary cap violators and the remaining 312 teams as non-violators. The average age for these rosters was 26.20 years (*SD* = 1.55; Range: 22.60–31.20 years). On average, and as expected, the sampled teams won 50.00% of their games (*SD* = 14.85%; Range: 10.60%–89.02%). During the sample period, the average number of team possessions per game was 98.00 (*SD* = 3.38; Range: 89.76–106.01). Teams featured in our sample had an average offensive rating of 108.59 (*SD* = 4.74; Range: 94.40–122.20). The mean defensive rating across sampled teams was 108.60 (*SD* = 4.44; Range: 97.50–119.60). Lastly, teams played in an average market size comprised of an estimated population of 5,563,841.05 (*SD* = 4,823,636.93), with the smallest market having an estimated population equal to 962,165 and the largest being 20,048,886 people. Figure 1 offers visualizations of the sample statistics.

### 4.2 Point-biserial correlations

**Figure 2:**
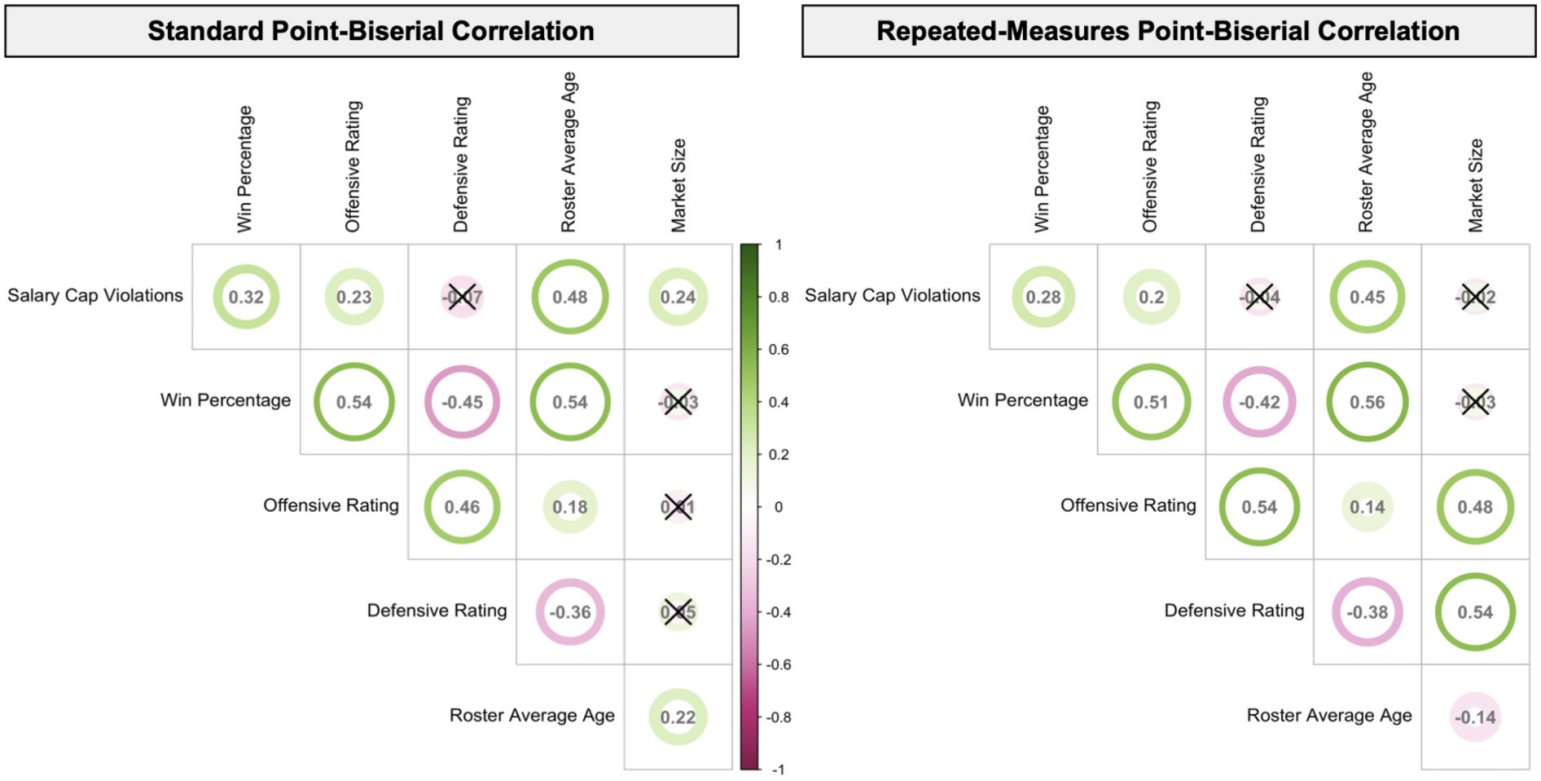
Correlogram displaying point-biserial correlations between salary cap violations, main outcomes, and covariates. Color gradient represents the strength of correlation with green as positive and pink as negative. Coefficients are reported within each circle. The outline around each circle represents the 95% confidence interval. Unless otherwise noted, all correlations are significant at the *p* < .001 level. Non-significant correlations are marked with an ×.

According to the standard point-biserial correlations, salary cap violations were positively correlated with win percentage, offensive rating, roster average age, and market size (all *r_pb_* values > 0.20, all *p*-values < .001). These correlations suggest that teams that violated the salary cap tended to have higher such values of these variables, with roster average age showing the strongest such relationship (*r_pb_* = 0.48, *p* < .001). By and large, the direction and strength of the within-team associations between these outcomes remained similar. However, the relationship between salary cap violation and market size was not evident within teams (*r_rm_* = −0.02, *p* = .68). Figure 2 provides a visual summary of the correlations.

### 4.3 Mixed-effects models

**Figure 3:**
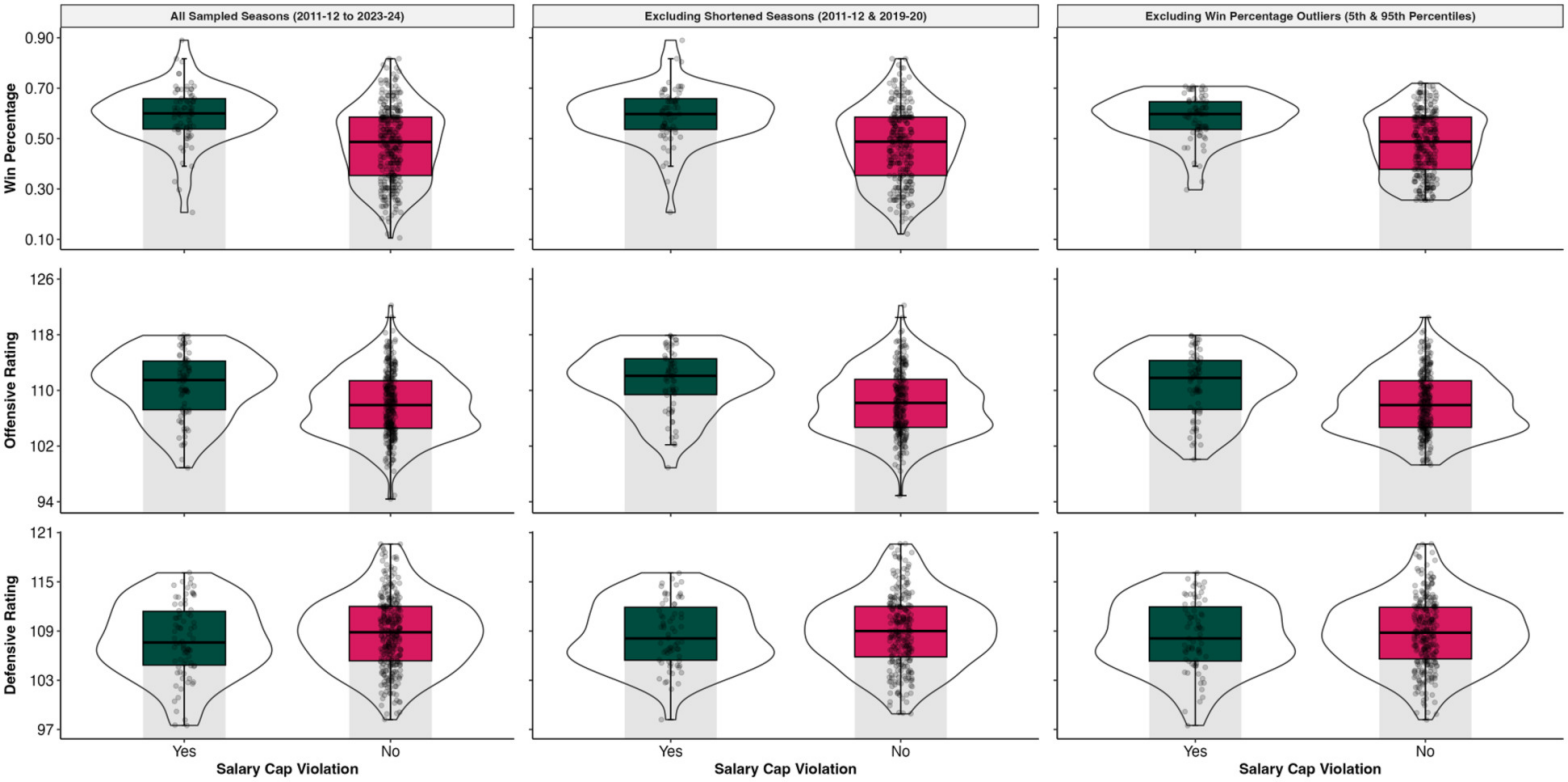
Pirateplots for win percentage, offensive rating, and defensive rating by model.

As evidenced by the marginal and conditional *R*^2^ values, all models experienced an appreciable improvement in model fit upon inclusion of the random effects for team and season (*R*^2^_Marginal_ range: .04 –.33; *R*^2^_Conditional_ range: .36 –.75). Overall, the mixed-effects models including all sampled seasons revealed no significant differences in performance between salary cap violators and non-violators at the *α* = .05 level (see Table 1). Figure 3 provides an illustration of the continuous outcomes using pirateplots. However, inspection of the model results did reveal some marginal differences (*p* < .10). Teams that violated the salary cap [Estimated Marginal Mean [*EMM*] = 70.19%, Standard Error [*SE*] = 8.12] appeared to have a marginally higher probability (*p* = .07) of making the playoffs compared to non-violators (*EMM* = 52.40%, *SE* = 4.98%). Specifically, salary cap violators had 2.14 times the odds of making the playoffs than non-violators [95% CI: (0.95, 4.81)]. However, upon exclusion of the 2011–2012 and 2019–2020 shortened seasons, these marginal differences appeared to attenuate, as characterized by a decline in the odds ratio and higher *p*-values.

**Table 1:** Mixed-effects model results.

Model	Outcome	Main variable			Covariates									Model summary
		Salary cap violation			Market size			Roster average age			Roster average age (Squared)			
		*b*	ES	*p*	*b*	ES	*p*	*b*	ES	*p*	*b*	ES	*p*	*R* ^2^_M_
		(*SE*)	[95% CI]		(*SE*)	[95% CI]		(*SE*)	[95% CI]		(*SE*)	[95% CI]		(*R*^2^_C_)
All sampled seasons (2011–2012 to 2023–2024)^a^	Playoff status	−0.76 (0.41)	0.47 [0.21, 1.05]	.07	−0.25 (0.19)	0.78 [0.54, 1.13]	.18	1.24 (0.18)	3.47 [2.42, 4.99]	***	−0.01 (0.14)	0.99 [0.76, 1.29]	.94	.33 (.42)
	Win percentage	−0.03 (0.02)	−0.03 [−0.06, 0.01]	.15	−0.02 (0.01)	−0.02 [−0.05, −0.004]	.02	0.08 (0.01)	0.08 [0.07, 0.10]	***	−0.01 (0.005)	−0.01 [−0.02, 0.002]	.15	.32 (.43)
	Offensive rating	−0.77 (0.42)	−0.77 [−1.60, 0.05]	.06	−0.43 (0.25)	−0.43 [−0.92, 0.05]	.08	1.52 (0.18)	1.52 [1.17, 1.86]	***	−0.25 (0.11)	−0.25 [−0.46, −0.04]	.02	.09 (.73)
	Defensive rating	−0.29 (0.37)	−0.29 [−1.01, 0.44]	.44	0.39 (0.23)	0.39 [−0.06, 0.84]	.09	−1.28 (0.16)	−1.28 [−1.59, −0.98]	***	0.03 (0.09)	0.03 [−0.16, 0.21]	.77	.07 (.73)
Excluding shortened seasons (2011–2012 & 2019–2020)^b^	Playoff status	−0.64 (0.45)	0.53 [0.22, 1.28]	.16	−0.31 (0.19)	0.73 [0.51, 1.07]	.10	1.21 (0.20)	3.35 [2.26, 4.98]	***	0.05 (0.15)	1.05 [0.79, 1.41]	.73	.33 (.40)
	Win percentage	−0.02 (0.02)	−0.02 [−0.06, 0.02]	.23	−0.03 (0.01)	−0.03 [−0.05, −0.01]	.01	0.08 (0.01)	0.08 [0.07, 0.10]	***	−0.01 (0.005)	−0.01 [−0.02, 0.004]	.21	.32 (.42)
	Offensive rating	−0.76 (0.48)	−0.76 [−1.71, 0.18]	.11	−0.48 (0.26)	−0.48 [−0.99, 0.04]	.07	1.58 (0.20)	1.58 [1.19, 1.96]	***	−0.20 (0.12)	−0.20 [−0.43, 0.03]	.09	.10 (.73)
	Defensive rating	−0.44 (0.42)	−0.44 [−1.26, 0.38]	.29	0.45 (0.22)	0.45 [0.02, 0.88]	.04	−1.26 (0.17)	−1.26 [−1.60, −0.92]	***	0.10 (0.10)	0.10 [−0.10, 0.30]	.34	.07 (.72)
Excluding win percentage outliers (5th & 95th percentiles)^c^	Playoff status	−0.82 (0.43)	0.44 [0.19, 1.02]	.06	−0.17 (0.19)	0.84 [0.58, 1.21]	.35	1.04 (0.18)	2.82 [1.97, 4.03]	***	−0.03 (0.13)	0.98 [0.76, 1.26]	.85	.27 (.36)
	Win percentage	−0.04 (0.02)	−0.04 [−0.07, −0.005]	.03	−0.02 (0.01)	−0.02 [−0.04, 0.002]	.09	0.06 (0.01)	0.06 [0.05, 0.07]	***	−0.01 (0.004)	−0.01 [−0.02, <.001]	.05	.26 (.38)
	Offensive rating	−0.89 (0.42)	−0.89 [−1.72, −0.06]	.04	−0.24 (0.24)	−0.24 [−0.71, 0.23]	.31	1.13 (0.18)	1.13 [0.78, 1.47]	***	−0.25 (0.11)	−0.25 [−0.47, −0.04]	.02	.06 (.75)
	Defensive rating	0.01 (0.38)	0.01 [−0.73, 0.75]	.97	0.29 (0.22)	0.29 [−0.14, 0.71]	.19	−0.89 (0.16)	−0.89 [−1.20, −0.58]	***	0.10 (0.10)	0.10 [−0.09, 0.29]	.29	.04 (.75)

^a^ *n* = 390 observations across 13 seasons.

^b^ *n* = 330 observations across 11 seasons.

^c^ *n* = 352 observations across 13 seasons. Salary cap non-violators used as the reference group. Covariates were mean-centered for analyses. *b* = unstandardized coefficient. SE = standard error. Odds ratio reported as effect size (ES) for models analyzing playoff status. Beta coefficient reported as ES for all other models. *R*^2^M, marginal *R*^2^; *R*^2^C, conditional *R*^2^.

*** *p* < .001.

Sensitivity analyses excluding outliers in win percentage (i.e., teams past the 5th and 95th percentiles) yielded compelling evidence to suggest significant changes across outcomes. Teams that violated the salary cap won significantly more games (*p* = .03; EMM = 53.08%, SE = 1.65%) and had higher offensive ratings (*p* = .04; EMM = 109.33, SE = 1.14) than those that did not violate the salary cap (Win Percentage: EMM = 49.33%, SE = 1.01%; Offensive Rating: EMM = 108.46, SE = 1.09). The beta coefficient from the analysis of win percentage showed an increase of 0.04 standard deviation for teams that violated the salary cap [95% CI: (0.005, 0.07)]. In contrast, there was a remarkably larger increase of 0.89 standard deviation in offensive rating for salary cap violators [95% CI: (0.06, 1.72)]^1^. Notably, the linear effect of the age covariate was significant across all models (all *p*-values < .001). However, the quadratic term for age was significant only for the models that included all sampled seasons and excluded win percentage outliers.

## 5 Discussion and conclusion

The hypothesis that investing in more expensive rosters justifies financial fines for violating the salary cap due to the ensuing improvement in team performance was tested using NBA teams' data spanning 13 seasons from 2011–2012 to 2023–2024. Differences among salary cap violators and non-violators for season win percentages, playoff status, and offensive and defensive rating were examined using a series of mixed-effects models with random effects for team and season. Although salary cap violators appeared to have higher odds of making it to playoffs and higher offensive ratings, the differences with non-violators are marginal and not statistically significant when using the full sample, as well as upon exclusion of the 2011–2012 and 2019–2020 shortened seasons. However, with outliers—teams outside the 5th and 95th percentiles of win percentage—excluded, the results yielded more compelling evidence to suggest that violators may win more games and have higher offensive ratings than non-violators. Thus, investing in more expensive rosters may justify the imposition of luxury tax penalties for exceeding the salary cap, as this leads to an improvement in performance for most teams, except for the very best and the worst performing teams.

For instance, consider the case of the 2017–2018 Toronto Raptors, who finished the regular season with a record of 59–23 (Win Percentage: 71.95%; 95.12 percentile during the sample period) and the first seed in the Eastern Conference, with no luxury tax bill and a team payroll that ranked outside the top 10 in the NBA. In our analyses, we deemed their performance as outlying and excluded their season in our sensitivity analyses. However, our study did not consider teams' eventual playoff performances, as this iteration of the Raptors went on to be swept by the Cleveland Cavaliers in the second round of the NBA playoffs (30). During the offseason, the Raptors fired their head coach, Dwane Casey, who had just won Coach of the Year (31). The team also made a significant trade ahead of the 2018–2019 season. In exchange for DeMar DeRozan, Jakob Poetl, and a protected 2019 first-round draft pick, the Raptors acquired Kawhi Leonard and Danny Green (32). However, following this trade, the team payroll had ballooned past the salary cap and their luxury tax hit the third highest in the league.

Following the subsequent 2018–2019 season, the team's performance mirrored the previous year, as the Raptors finished with a 58–24 record (Win Percentage: 70.73%; 94.10 percentile during the sample period) but dropped to the second seed in the East. This season fell just under the threshold for exclusion and was retained in our sensitivity analyses. Consequently, the Raptors' investment in their roster yielded the ultimate success, culminating in their victory of the 2019 NBA Finals, with Kawhi Leonard named the Finals MVP. Regrettably, the team was unable to sustain this level of performance in the ensuing seasons, as Leonard departed the team for the Los Angeles Clippers during the offseason (33). Thus, a team without a luxury tax liability, such as the Raptors, could seize an opportunity to increase its chances of not only qualifying for the playoffs but possibly winning a championship by fully committing to enhancing and investing in its roster. However, although this strategy may prove beneficial in the short-term, NBA contracts span multiple years, and consistently exceeding salary caps could restrict a team's long-term flexibility.

In turn, one limitation of the current study is that we examined a static situation, as both salary cap status and team performance are measured within the same season. This approach may not fully capture the potential lagged effects of salary cap violations. It is plausible that violating the salary cap could have long-term effects on future team performance, which the current study does not account for. Specifically, the analyses overlook any changes in team performance between seasons. A longitudinal study is necessary to capture these long-term effects of salary cap violations on performance across future seasons. Moreover, the current analysis centers solely on on-court performance and neglects the potential off-court advantages associated with salary cap violations. It may be that salary cap violators experience off-court benefits, such as increased revenues through greater merchandise sales and higher home game attendance, due to the presence of superstar players on their roster. Incorporating data on off-court metrics would provide a better understanding of the broader implications of salary cap violations and would extend the results of the current and existing research.

It is also possible that a more subtle relationship between salary cap violation and performance can exist. For example, these effects might be more prominent among teams playing in smaller (or, conversely, larger) markets or for younger teams. That is, teams in larger markets may be better positioned to absorb luxury tax penalties and attract high-caliber talent, while smaller market teams might face more pronounced negative consequences. Consequently, models that incorporate interaction terms between variables or operationalize market size in a different manner (e.g., categorically) could potentially tease out these effects. We also acknowledge that our sample period, which spanned from the most recent lockout during the 2011–2012 season until the 2023–2024 season, might have constrained our statistical power. Thus, including additional seasons could have provided stronger evidence of differences between salary cap violators and non-violators.

Moreover, in 2023, the new Collective Bargaining Agreement (CBA) modified the salary cap system by introducing additional cap thresholds, termed *aprons*. A team whose salary exceeds the first apron is prohibited from making certain moves during that league year (e.g., acquiring players via sign-and-trade requires reducing team salary under the apron, teams must match salaries within specific limits during trades, and teams cannot sign players waived during the regular season above the midlevel exception), while a team whose salary goes beyond the second apron faces even more restrictions [e.g., not being able to use midlevel or trade exceptions; (34)]. Teams under the previous CBA were granted much more flexibility due to the salary cap system operating with tiered thresholds that imposed progressively higher luxury taxes. The primary objective of introducing aprons is to encourage competitive balance by limiting the ability of the teams with the NBA's highest payrolls to further upgrade their rosters (35). However, the full restrictions imposed by the newly implemented two-apron system did not become effective until the 2024–2025 season (36). Thus, although we opted to include the 2023–2024 season in our dataset, its inclusion may be warranted considering the phased rollout of the new system. In addition, we refrained from removing this season from our truncated model, which excluded shortened seasons, due to the resulting further reduction in statistical power.

Although we did not consider the nuances of the current CBA, we encourage future research to investigate the impact of surpassing the first and, in certain instances, the second aprons on team performance and roster construction. For example, during the 2024–2025 NBA season, the league witnessed one of the most monumental trades in its history, involving Luka Dončić of the Dallas Mavericks who was traded to the Los Angeles Lakers. Dončić, one of the league's top superstars, who had recently led the Mavericks to the NBA Finals the previous season, was eligible for a 5-year, $345 million contract extension. This contract would have been the most lucrative in NBA history (37). Some reports speculate that this trade might have been partly motivated by the two-apron system. Thus, while the NBA witnessed teams willing to venture deep into the luxury tax in previous years, teams may now exhibit a newfound reluctance to enter the first and second aprons to maintain roster flexibility, particularly during the regular season. It appears that even large market teams may not be immune to this strategy, as evidenced by the Mavericks, who consistently ranked among the top seven in market size during the sample period.

Ultimately, to further evaluate the efficiency of team spending, future research could utilize non-parametric statistics, such as data envelopment analysis (DEA), which estimates the efficient frontier using linear programming techniques proposed by Charnes et al. (38). DEA is particularly well-suited for assessing multi-input, multi-output decision-making environments, such as professional sports teams. For instance, Yang et al. employed DEA to evaluate the efficiency of NBA teams, decomposing overall team efficiency into two components: first-stage wage efficiency and second-stage on-court efficiency. Their findings revealed that NBA teams excelled in wage efficiency compared to on-court efficiency, as on-court performance is influenced by numerous uncontrollable factors (e.g., officiating). The results also suggest that general managers can enhance organizational efficiency by recruiting players that better fit the team (39). Building upon that paper, Chatzistamoulou et al. add the salary cap (but not its violation) as one of the inputs in their DEA approach. Their findings reveal that experienced teams realized improved performance, while organizational gaps declined (7). Future DEA-based studies could incorporate salary cap violations as another input, therefore providing more granular insights into how these financial decisions impact team efficiency. Thus, a deeper understanding of salary cap allocation, particularly weighing the costs and benefits of entering the luxury tax, and now the first and/or second aprons, is vital to the success of NBA teams. By analyzing the various effects of violations among teams, general managers and practitioners can more efficiently construct team rosters that improve their chances of securing playoff berths and contending for championships.

## Data Availability

Publicly available datasets were analyzed in this study. This data can be found here: Basketball-Reference (https://www.basketball-reference.com/) and Spotrac through a Premium Account (https://www.spotrac.com).

## Footnotes

^1^The results from the sensitivity analyses remained consistent even after excluding data from the shortened seasons (2011–2012 and 2019–2020), along with teams with win percentages beyond the 5th and 95th percentiles.
